# Highlight: Reconciling Differences between Germline and Soma

**DOI:** 10.1093/gbe/evab102

**Published:** 2021-06-30

**Authors:** Casey McGrath

This month, *Genome Biology and Evolution* publishes its first Special Section, a group of papers on the subject of “Within-individual genome variation and germline/soma distinction.” The section includes six articles/works highlighting current research and emerging trends in the burgeoning field of within-individual genomic analyses. Headlining the Special Section is a Perspectives piece written by guest editors Anne-Marie Dion-Côté of the University of Moncton and Alexander Suh of the University of East Anglia and Uppsala University (Suh and Dion-Côté 2021).

The Special Section evolved out of a symposium that was originally proposed by Dion-Côté, Suh, and *GBE* editor-in-chief Laura A. Katz of Smith College, as part of the Society for Molecular Biology and Evolution’s planned 2020 conference. Credit for the topic idea goes to Dion-Côté: “Working on germline-restricted chromosomes as a postdoc opened my eyes to the diversity of mechanisms by which genomes are rearranged, edited, and amplified within an individual across taxa. I really wanted to showcase this surprising diversity that is rarely taught and build long-lasting links across members of the small community studying these mechanisms.” When the SMBE conference was canceled due to COVID-19, the organizers held a virtual event that included talks by the original speakers slated to appear at the symposium. The articles in this Special Section were contributed by some of the speakers and organizers of this virtual meeting.

The Special Section focuses on differences between germline genomes (those that will be transmitted to the next generation) and somatic genomes (those that are not inherited). In humans, for example, the genomes of the egg and sperm represent germline genomes, as do the ovary and testis cells giving rise to these, whereas those in every other cell in the body are somatic. Differences among genomes within the same individual can stem from a number of mechanisms, including mutation during DNA replication, DNA damage repair, the activity of transposable elements, or somatic recombination, which is used to generate genetic variation for antibodies.

One process that may result in genomic differences between the germline and soma is programmed DNA elimination (also called chromatin diminution). In a range of organisms including unicellular ciliates and various animals such as nematodes and copepods, specific genomic sequences are selectively eliminated from developing somatic nuclei. In hagfishes, lampreys, songbirds, and some arthropods, entire chromosomes are eliminated. Only recently has sequencing data become available for some of these organisms, confirming that many germline-restricted chromosomes contain genes that are expressed in germline cells. In the current Special Section, a review by Hodson and Ross (2021) highlights evolutionary perspectives on germline-restricted chromosomes in three families of flies, each thought to have evolved this feature independently. Unfortunately, few genomic data are available for these lineages. In particular, sequences belonging to germline-restricted chromosomes can be difficult to identify within genomic data. In an attempt to facilitate this process, Asalone et al. (2021) used a comparative coverage pipeline normally used for transcriptomic analysis to enable the discovery of germline-restricted sequences in zebra finch, adding to the available genomic toolkit for identifying such sequences.

In addition to programmed DNA elimination, differences between germline and somatic genomes can be generated by uniparental genome elimination, in which the entire complement of chromosomes inherited from one parent is removed/deleted from the germline. This happens during hybridogenesis, a process known to occur in some plants and frogs. Majtánová et al. (2021) reveal that Australian carp gudgeon hybrids also undergo genome elimination: one parental genome is selected for transmission and is duplicated in the germline cells of juvenile carp gudgeons, whereas the other is eliminated.

Within-individual genomic variation can also arise from differences among organellar, rather than nuclear, genomes. In most animals, the mitochondrial genome is only inherited from a single parent, limiting the potential for variation. In over 100 bivalve species, however, highly distinct forms of mitochondria are inherited from each parent. In particular, the paternally inherited mitochondrial form undergoes a tight bottleneck in each generation, as only four to five mitochondria are present in each sperm cell. In their article, Iannello et al. (2021) analyzed the genomic variability among mitochondria within individuals and showed that this narrow bottleneck resulted in extremely high variability among mitochondria across tissues.

Finally, although most studies of germline/soma differences have focused on eukaryotes, such distinctions can even be found among prokaryotes. In her review, Angert (2021) looks at variation among inherited and noninherited chromosomes in the highly polyploid bacterial lineage *Epulopiscium* sp. type B. In these cells, only 1% of the genetic material in a mother cell is directly inherited by offspring cells, leading chromosomes to take on a somatic or germline role. Angert’s review highlights that recombination is vital to diversification in populations of these prokaryotes.

Together, the articles in this Special Section represent an important contribution to the field of within-individual genomic variation. According to the Perspectives piece by Suh and Dion-Côté (2021), the new findings described in these articles have been made possible in part by the development of more efficient methods of high-throughput sequencing. In the future, they “anticipate that the continuous improvement of sequencing read length and quality will further increase the resolution for detecting different types of somatic variation.” Dion-Côté and Suh also pose several outstanding questions for the field. A key question is how common various forms of within-individual genome variation are across various lineages. According to Suh, surprisingly little is known about the prevalence of such variation. “It still baffles me how few organisms have so far been looked at for any form of within-individual genome variation. Songbirds, for example, are one of the best-studied groups of organisms in many fields. Yet, even though germline-restricted chromosomes were first identified in zebra finch over two decades ago, it was only very recently shown that such chromosomes are likely present in all ∼5,000 songbird species. It’s surprising that this could have been overlooked for so long.” Suh and Dion-Côté share a hope that, in the future, researchers will start looking for within-individual genome variation across a wider range of organisms to reveal whether “germline/soma genome differences are the odd exception or the overlooked rule across the tree of life.” Although a lack of awareness on intraindividual genome variation may have been a barrier to this in the past, Dion-Côté hopes that this Special Section will “showcase how widespread and potentially important germline/soma differences might be.”
